# Supplementary material for: The causal relationship between the human gut microbiota and pyogenic arthritis: a Mendelian randomization study
Source: Front Cell Infect Microbiol. 2024 Nov 26;14:1452480. doi: 10.3389/fcimb.2024.1452480 (PMC11629706; doi:10.3389/fcimb.2024.1452480)
Supplement: Supplementary file 1 [file DataSheet1.zip › mendelian test/ebi-a-GCST90027540.csv_forest.pdf]

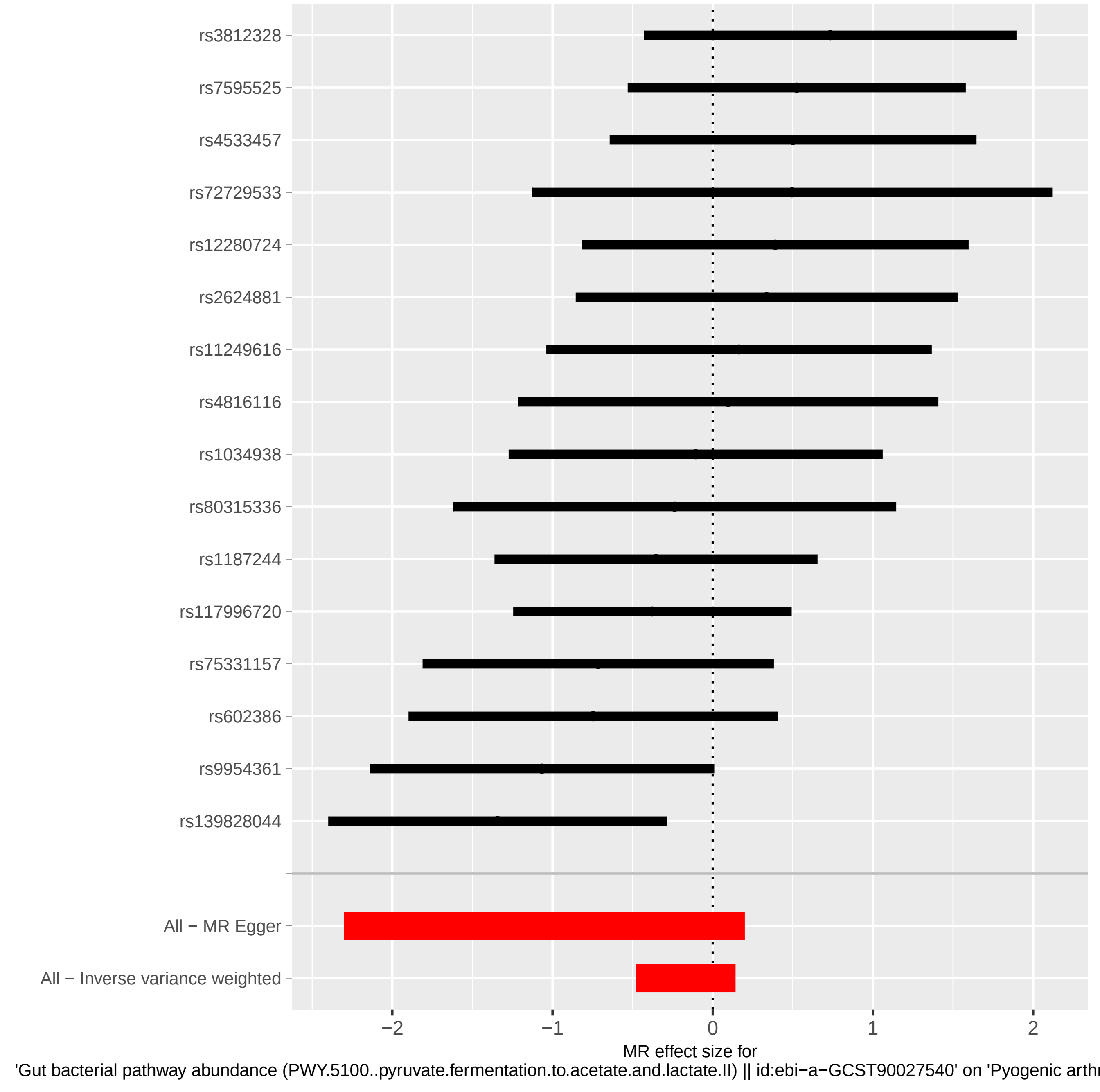

rs3812328
rs7595525
rs4533457
rs72729533
rs12280724
rs2624881
rs11249616
rs4816116
rs1034938
rs80315336
rs1187244
rs117996720
rs75331157
rs602386
rs9954361
rs139828044
All − MR Egger
All − Inverse variance weighted
−2
−1
0
1
2
MR effect size for
'Gut bacterial pathway abundance (PWY.5100..pyruvate.fermentation.to.acetate.and.lactate.II) || id:ebi−a−GCST90027540' on 'Pyogenic arth
